# Synthesis of coaxial carbon@NiMoO_4_ composite nanofibers for supercapacitor electrodes†

**DOI:** 10.1039/c8ra05912h

**Published:** 2018-09-24

**Authors:** Changqing Teng, Xuehui Gao, Ning Zhang, Yu Jia, Xiaoyu Li, Zhengyu Shi, Zongxiao Wu, Mingjia Zhi, Zhanglian Hong

**Affiliations:** State Key Laboratory of Silicon Material, School of Materials Science and Engineering, Zhejiang University 38 Zheda Road Hangzhou 310027 China Mingjia_zhi@zju.edu.cn Hong_zhanglian@zju.edu.cn

## Abstract

This work reports the synthesis of coaxial carbon@NiMoO_4_ nanofibers for supercapacitor electrode applications. Thin NiMoO_4_ nanosheets are uniformly coated on the conductive electrospun carbon nanofibers by a microwave assisted hydrothermal method to form a hierarchical structure, which increases the porosity as well as the conductivity of the electrode. The thickness of the NiMoO_4_ can be easily adjusted by varying the precursor concentrations. The high specific surface area (over 280 m^2^ g^−1^) and conductive carbon nanofiber backbone increase the utilization of the active pseudocapacitive NiMoO_4_ phase, resulting a high specific capacitance of 1840 F g^−1^.

## Abstract 2

This work reports the synthesis of coaxial carbon@NiMoO_4_ nanofibers for supercapacitor electrode applications.

## Graphical abstract

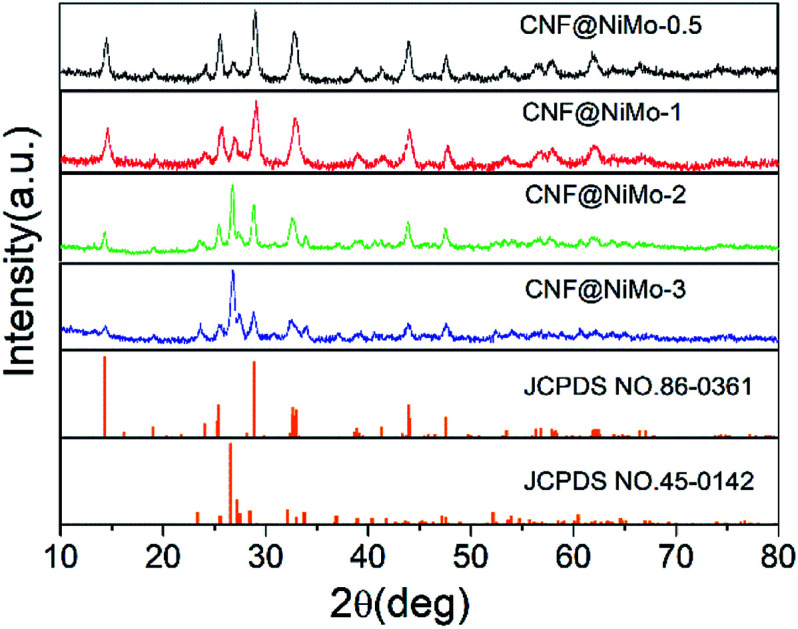

## 1. Introduction

In recent years, transitional metal oxides, especially binary and ternary metal oxides, have attracted much interest for supercapacitor electrodes, since they can offer higher energy density than typical carbonaceous materials.^1,2^ The superior performance of these complex oxides originated from the higher number of multiple oxidation states than that in the single component metal oxides. Among them, NiMoO_4_ is believed to be one of the most proposing candidates, as it has high specific capacitance. The main charge storage capability comes from the high electrochemical activity of Ni.^4^

Nevertheless, like other metal oxides, NiMoO_4_ also suffers from the drawbacks of poor electronic conductivity, which may hinder its application in high-power density supercapacitors. In addition, increasing the numbers of the surface redox sites is crucial for high energy density purpose. One strategy is to tune the microstructure of the pristine NiMoO_4_ materials. Different types of NiMoO_4_ nanostructures such as nanorods,^4–8^ nanospheres,^5^ nanosheets^9–12^ and nanotubes^13^ have been prepared so far. High specific capacitances were also reported. For instance, hierarchical NiMoO_4_ nanosheets were directly grown on Ni foam by a facile hydrothermal and deliver the specific capacitance of 1221 F g^−1^ at 1 A g^−1^.^9^ NiMoO_4_ nanospheres had a high specific capacitance of 974 F g^−1^.^5^

Another possible solution would be incorporating NiMoO_4_ with conductive substrate, especially carbon nanostructures, which may improve the charge transfer capability of the composite and enable increasing the specific surface area.^14–18^ The NiMoO_4_/rGO composite electrode showed 1274 F g^−1^ capacitance at 1 A g^−1^, due to the enlarged specific surface area by coupled with conductive rGO.^19^ The key requirements for constructing these structures lay on the porous nature of the carbon nanomaterials, as well as the intimate coupling between NiMoO_4_ and the carbon interface. Such rational designing would not only improve the electrical conductivity, it even shortens the ions diffusion path.^20–22^

In this work, coaxial carbon/NiMoO_4_ composite nanofibers are prepared. In such composite, thin NiMoO_4_ nanosheets are grown outwards on the conductive electrospun carbon nanofibers (CNFs) to form the coaxial porous structure, in which the carbon fibers serve as the core and NiMoO_4_ nanosheets as the shell. The nanosheets form an intimate connection with the CNFs, which benefits the electron transfer and enlarges the redox active surface. Also, the porous CNFs backbone facilitates the electrolyte penetration into the composite, which is desirable for improve the rate capability. As a result, the prepared CNF@NiMoO_4_ has a large surface area of over 280 m^2^ g^−1^ and manifests faradaic capacitance of 1840 F g^−1^ (based on the mass of active NiMoO_4_). The asymmetric capacitor based on the CNF@NiMoO_4_ cathode can deliver a high energy density of 23.9 W h kg^−1^ at a power density of 0.75 kW kg^−1^ (the calculation is based on the whole electrode, including the mass of NiMoO_4_ and CNFs) and it shows good cycling stability with the retention of 78.3% after 10 000 cycles.

## 2. Experimental

### 2.1. Synthesis of the carbon nanofibers backbones

The carbon nanofibers were fabricated by electrospinning PAN (polyacrylonitrile)–DMF (*N*,*N*-dimethylformamide) precursor and followed by carbonization process.^23–25^ The electrospinning precursor was prepared by dissolving 1.2 g of PAN in 10 ml of DMF at 60 °C under vigorous stirring. 0.6 g of iron(iii) acetylacetonate was then added into the precursor. According to the literature, the addition of Iron(iii) acetylacetonate into the precursor can significantly enhance the conductivity of CNFs.^26,27^ The precursor was subsequently loaded into an electro-spinner. A high voltage of 15 kV was adopted and the distance between the needle and plate collector was fixed at 15 cm. The flow rate was maintained at 1.0 ml h^−1^. The as-spun nanofibers were stabilized at 280 °C for 60 min in air and then carbonized at 800 °C for 2 h in N_2_ atmosphere. The carbonized nanofibers were then washed in HCl solution (1 mol l^−1^) for 24 h to remove any soluble Fe oxides. The obtained carbon nanofibers (CNFs) were cut into small pieces (1 × 1 cm^2^).

### 2.2. Synthesis of NiMoO_4_ @CNF

Ni(NO_3_)_2_·6H_2_O and Na_2_MoO_4_·2H_2_O was used to prepare NiMoO_4_ nanosheets. Typically, the chemicals were dissolved in the mixed solvent of H_2_O and ethanol (in 1 : 1 volume ratio, totally 30 ml) as the Ni–Mo precursor. The CNFs pieces were then immersed into the Ni–Mo precursor. After that, the suspension was heated in a sealed microwave reactor at 140 °C for 60 min. After the reaction chamber was cooled down to room temperature, the CNF@Ni–Mo precursor fibers were taken out and washed with DI water and ethanol for several times. Finally, the samples were heated at 450 °C for 120 min to obtain CNF@NiMoO_4_ composite nanofibers. Four different amounts of Ni–Mo precursors were applied and the samples were designated as CNF@NiMo-0.5, CNF@NiMo-1, CNF@NiMo-2 and CNF@NiMo-3. More detail information can be found in the ESI Table S1†. For comparison, pristine NiMoO_4_ was synthesized by the same procedure with CNF@NiMo-2.

### 2.3. Materials characterization

The crystal structure of the samples was characterized with X-ray diffraction (XRD, Cu-Kα irradiation; *λ* = 1.5418 Å). The morphology was observed by a scanning electron microscopy (SEM Hitachi S-4800) and transmission electron microscope (TEM; FEI Tecnai G2 F20 S-TWIN). The surface areas and the pore structures were measured by N_2_ adsorption/desorption method using Micrometric ASAP 2020 analyzer. The pore size distributions were calculated using the Barrett–Joyner–Halenda (BJH) method. The NiMoO_4_ loading amount was analysed by thermogravimetry (TG) and the samples were heated from room temperature up to 800 °C at a heating rate of 10 °C min^−1^ in air. The content of Fe in pure CNFs was analysed by ICP-OES (Optima 2100DV).

### 2.4. Electrochemical measurements

The electrochemical measurements were conducted using a three-electrode system in a 6 M KOH aqueous solution with a 760E electrochemical workstation (CH Instruments, Shanghai, China). The CNF@NiMo nanofiber mats were directly used as working electrodes, with Ag/AgCl as the reference electrode and Pt foil as the counter electrode.

The asymmetric supercapacitor device was fabricated with AC (activated carbon, Xianfeng Corp.) as the negative electrode and CNF@NiMo-2 as the positive electrode. The AC electrodes were prepared by mixing 80% activated carbon, 10% poly vinylidene fluoride (PVDF) binder, and 10% carbon black (super P) in *N*-methylpyrrolidinone (NMP), which was coated onto Ni foam as the negative electrode and dried at 60 °C for overnight.

## 3. Results and discussion

**Fig. 1:**
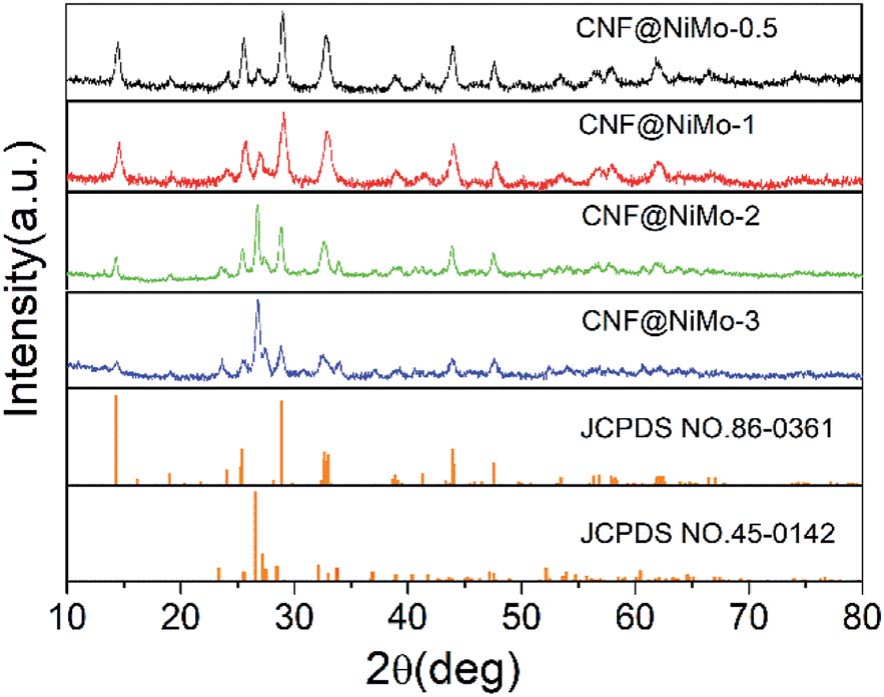
XRD pattern of different CNF@NiMo samples.

The crystalline structure of the CNF@NiMo has been characterized by XRD. Fig. 1 illustrates the XRD patterns of the different composite nanofibers. All the diffraction peaks indicate the successful formation of monoclinic structured nickel molybdenum oxide with a space group of *C*2/*m* (JCPDS card no. 86-0361 and 45-0142). The relative peaks intensities in the different samples are different. A higher concentration of Ni–Mo precursor leads to more JCPDS 45-0142 phase. This phenomenon subscribes to the previous paper, which reported the mixed phases can co-exist in the NiMoO_4_ nanostructures.^28^ The broad peaks also indicate that the size of crystalline grain of the NiMoO_4_ phase is small.

**Fig. 2:**
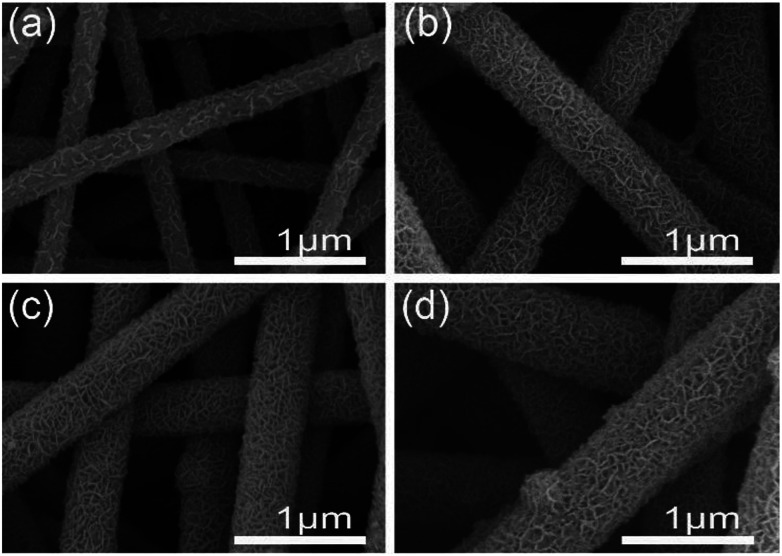
SEM images of (a) CNF@NiMo-0.5, (b) CNF@NiMo-1, (c) CNF@NiMo-2, and (d) CNF@NiMo-3.

The morphology of different CNF@NiMo samples has been analyzed by SEM and is shown in Fig. 2, and the morphology of raw CNFs (washed by HCl) can be found in Fig. S1.† From Fig. S1,† we can see that the surface of the raw CNFs is coarse, due to the etching of the Fe contained compounds.^26^ Such rough surface offers numerous nucleation and growth sites for NiMoO_4_. The diameter of the individual fiber is ∼400 nm and the length is over 30 μm. These nanofibers are integrated to form a porous structure. After the hydrothermal reaction and calcination, porous nanosheets appeared on the surface of the CNFs, which indicates the successful growth of NiMnO_4_ phase. Fig. 2(a) shows the sample with the lowest NiMo precursor concentration (CNF@NiMo-0.5), and it is evidently the squama like NiMoO_4_ textures can be found on the fiber surface. With the increasing of the NiMo precursor concentration, the NiMoO_4_ shell layer starts to cover the entire CNFs surface and becomes more homogeneous (Fig. 2(b–d)). By comparing the SEM images in Fig. 2, it can be founded that the surface of the composite nanofibers becomes more coarsened, and the diameter increases to ∼800 nm with the highest NiMoO_4_ loading. The observation above indicates that the NiMoO_4_ shell layer can be tuned by altering the precursor solution concentration.

**Fig. 3:**
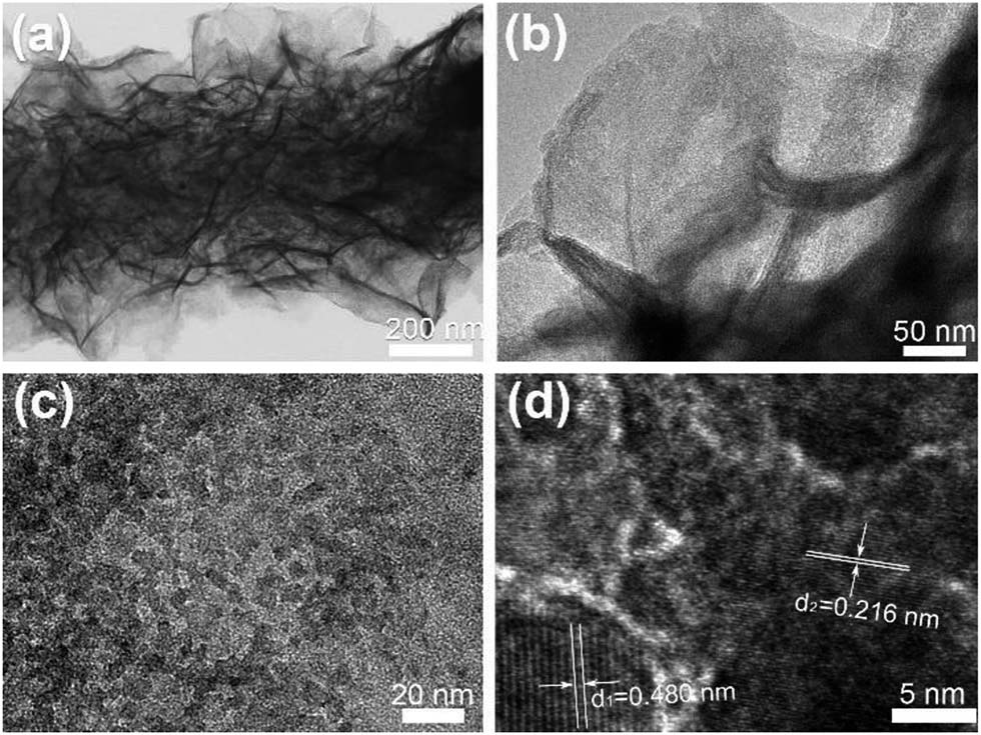
(a–c) TEM images of CNF@NiMo-2 at different magnification. (d) Corresponding HRTEM image of NiMoO_4_ nanosheets on CNFs.

TEM is further used to verify the porous nature of the composite nanofibers. Fig. 3 shows the TEM images taken from sample CNF@NiMo-2. Fig. 3(a) distinctly demonstrates the porous NiMoO_4_ layer has grown on a single CNF, which is constituted by numerous nanosheets. The layer thickness is approximate to the diameter of the CNFs. This result is in well agreement with the SEM observation. A closer view of the porous NiMoO_4_ layer in Fig. 3(b) confirms that the thickness of the nanosheets is about tens of nanometers, and the surface of nanosheets presents a great many of mesopores as shown in Fig. 3(c), which are mainly derived from the recrystallization process and the release of gas during the calcination process.^4,29^ In Fig. 3(d), the HRTEM image shows two kinds of lattice fringes with inter-planar distances of approximately 0.480 nm and 0.216 nm, respectively. These values are matched well with the spacing of the (−2 0 1) planes of JCPDS 86-0361 phase and the (2 2 2) planes of JCPDS 45-0142 phase. This result also accords well with the XRD analysis (Fig. 1).

The specific surface area and the pore structure are vital for supercapacitor electrodes. The enlarged surface area will increase the surface redox reaction sites, while the mesoporous structure will facilitate the ion transfer. The corresponding N_2_ adsorption/desorption isothermal curves is showed in Fig. S2†. All CNF@NiMo samples have the N_2_ isotherms close to type IV with steep uptakes below *P*/*P*_0_ = 0.01 and clear hysteresis loops, which indicates the coexistence of micropores (<2 nm) and mesopores(2–50 nm) in these samples.^18,30^ The porous feature guarantees the high specific surface areas of 335 m^2^ g^−1^, 319 m^2^ g^−1^, 283 m^2^ g^−1^, and 279 m^2^ g^−1^ in CNF@NiMo-0.5, 1, 2, and 3 respectively. When the amount of nanosheets is more intensive, the specific surface of CNF@NiMo composite fibers becomes slightly lower, possibly due to the thicker and denser NiMoO_4_ shell layer. The CNF@NiMo has the highest specific surface area in comparison with other NiMoO_4_ materials reported in the literature (Summarized in Table S2†), hence resulting in a high specific capacitance. The average pore diameter of CNF@NiMo-2 calculated from BJH method is 3.78 nm, which corresponds to mesopores observed in TEM analysis (Fig. 3(c)).

Fig. S3† shows the N_2_ adsorption/desorption isothermal curves and pore size distribution of the pristine CNFs. The specific surface area of the pristine CNFs is 208.7 m^2^ g^−1^, which is lower than that of the CNF@NiMo composites. The average pore diameter of the pristine CNFs is 6.10 nm, which is larger than those in the CNF@NiMo samples. The NiMoO_4_ nanosheets grown on the CNFs not only enlarge the surface area of the composite, but also reduce the pore size of CNF, since the whole surface of the CNF has been covered by the nanosheets.

In order to determine the exact NiMoO_4_ loading on CNFs, thermogravimetric analysis (TGA) has been used. The typical TGA curves of the different samples are illustrated in Fig. S4†. A sharp weight loss is observed in the pristine CNFs sample at the temperature of 410 °C, which indicates the combustion of carbon.^31^ The residue of the CNFs sample is mainly due to the Fe_2_O_3_ phase transformed from the iron acetylacetonate. The additional XRD (Fig. S5†) and EDS (Fig. S6†) analysis of the TG residue have proved this point. The CNF@NiMo samples also shows gradual weight loss between 300 °C to 500 °C, which is also due to the decomposition of carbon.^32^ The percentage of weight remaining (named *W*_r_) in CNF@NiMo-0.5, CNF@NiMo-1, CNF@NiMo-2, CNF@NiMo-3 and the pristine CNFs is about 34.9%, 37.4%, 40.6%, 49.0% and 13.6% after calcined at 800 °C in air. In the pristine CNFs samples, 13.6% of Fe_2_O_3_ is remained after the TG testing. For further confirming the content of Fe, ICP-AES has been adopted and shows that the Fe content is 9.71%, which is in accordance with TG analysis. Thus, the percentage of NiMoO_4_ mass (named *W*_m_) ratio can be calculated by the formula below:

1 *W*_m_ = (*W*_r_ − 13.6%)/(1 − 13.6%)

In which *W*_r_ refers to the weight remaining in the CNF@NiMo composite sample. Thus, the NiMoO_4_ mass ratio (*W*_m_) in CNF@NiMo-0.5, CNF@NiMo-1, CNF@NiMo-2, CNF@NiMo-3 is ∼24.6%, 27.6%, 31.3%, and 41.0%, respectively, which follows the order of the precursor concentration and is reflected in the shell thickness.

**Fig. 4:**
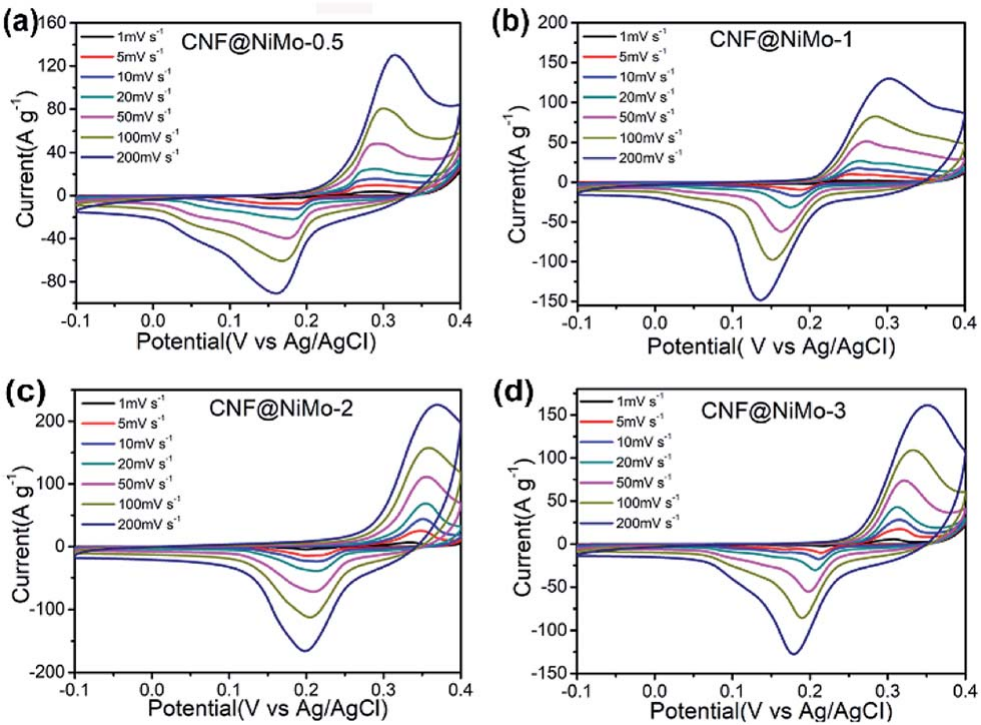
CV curves of (a) CNF@NiMo-0.5, (b) CNF@NMo-1, (c) CNF@NiMo-2, (d) CNF@NiMo-3 at various scan rates of 1–200 mV s^−1^.

The electrochemical performance of these CNF@NiMo composite nanofibers has been examined in three-electrode configurations. The typical cyclic voltammetry (CV) curves of four CNF@NiMo electrodes at different scan rates ranging from 1 to 200 mV s^−1^ are showed in Fig. 4. All the samples have the similar shapes of CV curves. The distinct pairs of redox peaks are observed in CNF@NiMo composite nanofibers within the potential range from 0.15 to 0.4 V *vs.* Ag/AgCl. That confirms the pseudocapacitive characteristics originating from the faradaic redox reactions of NiMoO_4_, which is associated to the following reaction as^4,33^

2 Ni(ii) ↔ Ni(iii) + e^−^

Apparently, the current intensity increases and the position of the redox peaks shifts slightly with the increased scan rates. Compared to the pristine NiMoO_4_ electrode (Fig. S7(a)†) or other published work,^9,19,28,34^ the shape of redox peaks still keeps integrity even the current intensity as high as 200 mV s^−1^, implying the better electrochemical reversibility. The CV curves of bare CNFs can be found in Fig. S8(a).† Clearly, the curves show the approximate rectangular shape and have a much smaller specific current density, and no distinct pairs of redox peaks are observed, which again indicates that the faradaic capacitance in the CNF@NiMo composite is mainly contributed from NiMoO_4_.

**Fig. 5:**
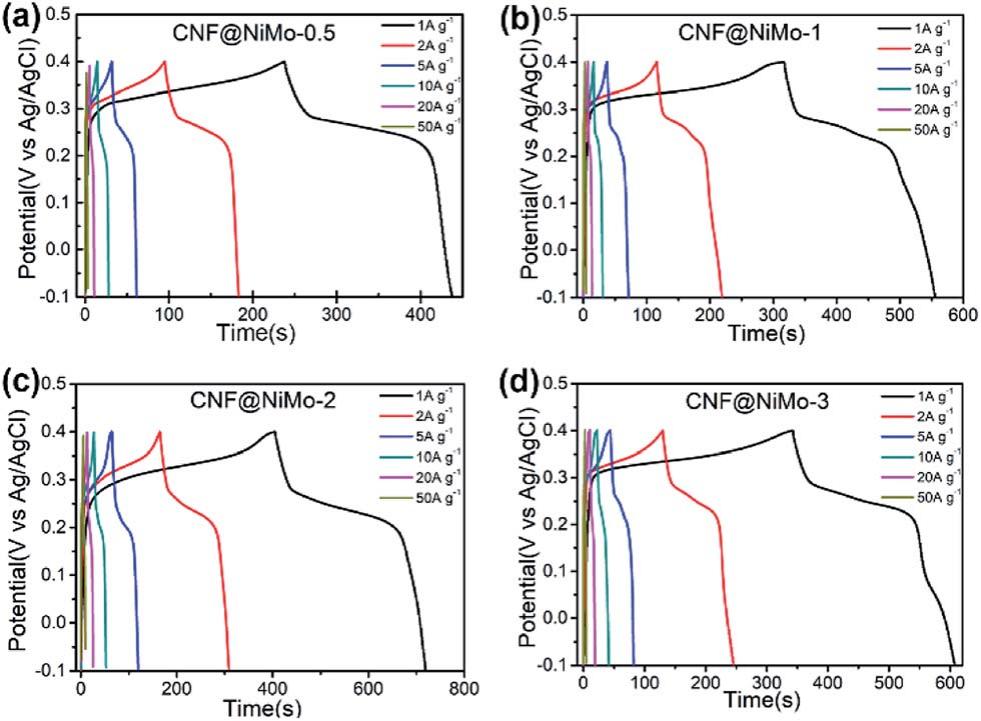
Galvanostatic charge/discharge voltage profiles of (a) CNF@NiMo-0.5, (b) CNF@NiMo-1, (c) CNF@NiMo-2, and (d) CNF@NiMo-3 electrodes in the voltage range of −0.1–0.4 V *vs.* Ag/AgCl.

**Fig. 6:**
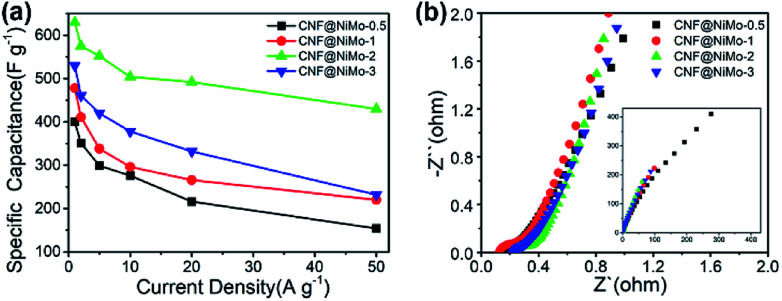
(a) The specific capacitance of different CNF@NiMo samples at various current densities, (b) electrochemical impedance spectra of different CNF@NiMo samples.

Fig. 5 depicts the charge/discharge voltage profiles of the different CNF@NiMo samples at current densities from 1 to 50 A g^−1^. The voltage window is −0.1–0.4 V *vs.* Ag/AgCl. The evident voltage plateaus in Fig. 5 further proves the pseudocapacitive behaviour of the electrode, which agrees well with the redox peaks observed in the CV curves. In contrast, the charge/discharge voltage profiles of the CNFs show a symmetrical triangle shape (Fig. S8(b), ESI†). No obvious pseudocapacitive behaviour can be observed in the pristine CNF electrode, which implies that the Fe content in the CNF has negligible effect on the capacitive behaviour of the composite electrode. This also states that the pseudocapacitive behaviour of CNF@NiMo electrode origins from NiMoO_4_ nanosheets rather than CNFs or Fe compound. The specific capacitance can be calculated by the formula:

3 *C* = *I*Δ*t*/*m*Δ*U*

where *I* is the discharge current, Δ*t* is the discharge time, Δ*U* is the voltage range (0.5 V), and *m* is the mass of the whole composite. Notably, the CNF@NiMo-0.5, 1, 2 and 3 electrodes deliver the specific capacitance of 400 F g^−1^, 478 F g^−1^, 630 F g^−1^ and 532 F g^−1^ at a current density of 1 A g^−1^(based on the mass of the whole electrode), and the specific capacitance can still maintain at 216 F g^−1^, 266 F g^−1^, 492 F g^−1^ and 332 F g^−1^ at a current density of 20 A g^−1^ as shown in Fig. 6(a). The CNF@NiMo-2 electrode shows the highest specific capacitance and the notable rate capability. In the meantime, the pristine CNFs shows the capacitance of 79 F g^−1^ at 1 A g^−1^ as in Fig S4(b).† Therefore the specific capacitance of the active NiMoO_4_ can be calculated by the following formula:^26^

4 *C*_(all)_ = *W*_(CNF)_ × 79 F g^−1^ + *W*_(NiMoO_4_)_ × *C*_(NiMoO_4_)_

It is furthermore noticeable to find that the CNF@NiMo-2 sample electrode delivers both the highest specific capacitance 1840 F g^−1^ at 1 A g^−1^ and rate capability of 78% at 20 A g^−1^(based on the mass of NiMoO_4_). However, the pristine NiMoO_4_ without CNFs, synthesized by the same procedure with CNF@NiMo-2, shows much lower specific capacitance of 1090 F g^−1^ at 1 A g^−1^ and rate capability of 59% at 20 A g^−1^(Fig. S7(b)†). It indicates that the combination of NiMoO_4_ and conductive CNFs does not only increase the specific capacity but also promote the rate performance. By comparison to the values from the reported NiMoO_4_ electrode materials (Table S2†), the CNF@NiMo-2 electrode possesses high specific capacitance and the outstanding rate capability at the same time. The impedance spectra of the electrodes support this finding and can be found in Fig. 6(b). The interception between the spectra and the *Z*′ axis at the high frequency range is used to obtain the equivalent series resistance. In the meantime, all the spectra show a step line at the low frequency range, which is the characteristic for capacitive electrode. The line of the CNF@NiMo-2 has the largest slope, confirming its good capacitive performance.

**Fig. 7:**
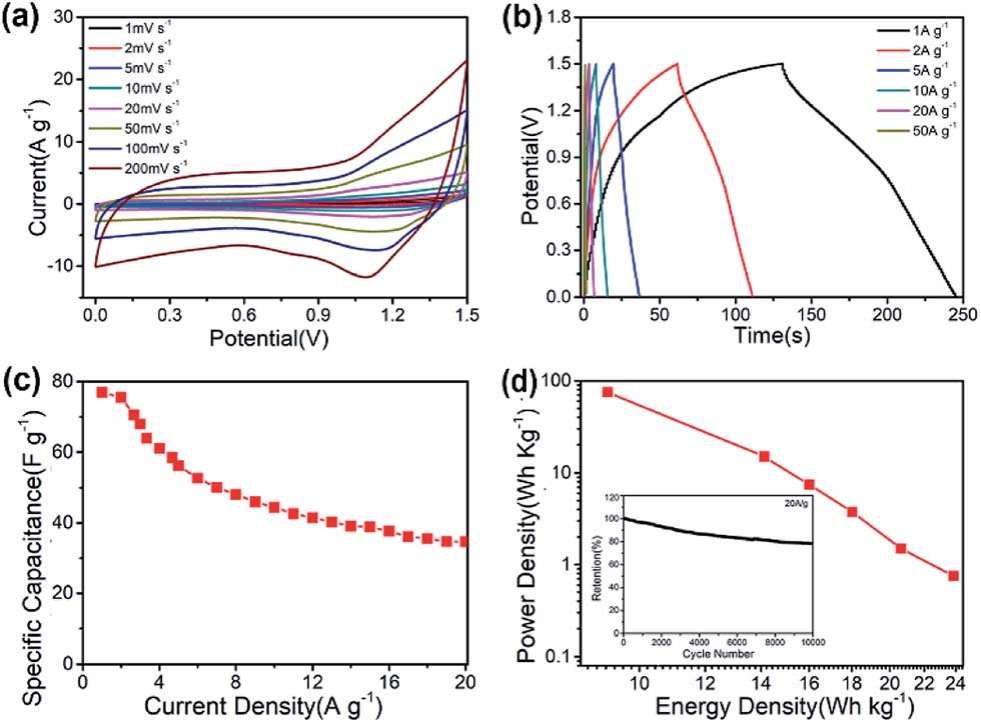
Electrochemical characterization of a CNF@NiMo-2//AC asymmetric supercapacitor device: (a) CV curves collected at different scan rates ranging from 1 to 200 mV s^−1^; (b) galvanostatic charge/discharge voltage profiles at different current densities in the voltage range of 0–1.5 V; (c) rate capability; (d) the power density of CNF@NiMo-2//AC ASC device at different energy density; The inset in (d) shows cycling stability at a current density of 20 A g^−1^.

A practical asymmetric supercapacitor device is assembled with CNF@NiMo-2 and AC (the CV curves and the Galvanostatic charge/discharge voltage profiles of AC are showed in Fig. S9†) as the positive and negative electrodes, respectively. The potential window is set to be 1.5 V. Fig. 7(a) shows the CV curves of the device at different scan rates (1 mV s^−1^ to 200 mV s^−1^). From the curve, the contribution from both electric double-layer capacitance and pseudo capacitance can be clearly observed. To illustrate the electrochemical properties of the asymmetric supercapacitor, galvanostatic charge/discharge curves at various current densities have been performed in Fig. 7(b). The curve shows quasi-triangle shape with good symmetry, indicating there is no water decomposition and the Faraday efficiency of the device is high. The specific capacitance based on the mass of whole two electrodes can reach a maximum of 76.4 F g^−1^ at a current density of 1 A g^−1^ and maintain at 35.6 F g^−1^ when the current density increases to 20 A g^−1^ (Fig. 7(c)). The long-term cycling performance of the CNF@NiMo-2//AC device is also studied, showed in Fig. 7(d). The CNF@NiMo-2//AC still maintained 78.3% after 10 000 cycles at a current density of 20 A g^−1^, suggesting the excellent electrochemical stability of the CNF@NiMo-2//AC device. Commonly, the energy density *E* and power density *P* are the two most important parameters for supercapacitor devices. At a power density of 0.75 kW kg^−1^, the CNF@NiMo-2 electrode delivers a high energy density of up to 23.9 W h kg^−1^, and still retains energy density of 11.2 W h kg^−1^ at high power density of 14.9 kW kg^−1^. The exceptional performance of CNF@NiMo-2//AC device could be attributed to its unique structure features. Specifically, hierarchical micro-nano structure allows for a synergistic integration of CNFs and NiMoO_4_. Functioned CNFs, the electrical conductivity of which is improved by Fe precursor,^35,36^ could provide electronic transmission channel,^37,38^ leading to enhanced rate performance; additionally, thin NiMoO_4_ sheets, with high specific surface area and much mesoporous, can deliver large specific capacity. Furthermore, macropores with much larger pore size have been constructed by the interconnected NiMoO_4_ nanosheets and CNFs framework, as evident from the SEM images shown in Fig. 2. This builds up a hierarchical pore structure composed of mesopores connected with macropores. In literature, it has been reported that the macropores can serve as solution buffering reservoir to minimize the diffusion distance, facilitate the mass transport, and also reduce the volume change during the charge/discharge cycling,^3–43^ ensuring a high cycling performance. Such hierarchical pore structure contributes to high energy density and power density of the composite electrode.

## 4. Conclusions

In summary, uniform NiMoO_4_ nanosheets arrays have been successfully grown on functional CNFs *via* rapid microwave-solvothermal method followed by thermal treatment. The thickness of the NiMoO_4_ shell layer can be easily tuned. The prepared CNF@NiMo-2 with 27.6% oxide content manifests superior faradaic capacitance of 1840 F g^−1^ and maintains 78% of its capacitance at 20 A g^−1^. An asymmetric supercapacitor with CNF@NiMo-2 as the positive electrode can operate at 1.5 V cell voltage and deliver a high energy density of 23.9 W h kg^−1^ at a power density of 0.75 kW kg^−1^. Good cycling stability with retention of 78.3% specific capacitance after 10 000 cycles is also achieved. The enhanced electrochemical performance of the CNF@NiMo-2 composite is mainly ascribed to the high specific surface of the uniform NiMoO_4_ nanosheets with mesoporous feature, as well as highly conductive CNFs. The strongly coupled composites may synergistically enhance the contact between electrolytes with active materials and shorten the ion diffusion path. Considering the desirable electrode configuration, easy fabrication process, and promising electrochemical performance, such a strategy might be readily extended for preparing other nanostructured electrode materials.

## Conflicts of interest

There are no conflicts to declare.

## Supplementary Material

RA-008-C8RA05912H-s001
