# Supplementary material for: Synthesis of coaxial carbon@NiMoO4 composite nanofibers for supercapacitor electrodes
Source: RSC Adv. 2018 Sep 24;8(57):32979–84. doi: 10.1039/c8ra05912h (PMC9086301; doi:10.1039/c8ra05912h)
Supplement: RA-008-C8RA05912H-s001 [file RA-008-C8RA05912H-s001.pdf]

Electronic Supplementary Material (ESI) for RSC Advances.
This journal is © The Royal Society of Chemistry 2018

Supporting Information

Table S1 the preparation details of the corresponding CNF@NiMo samples

Samples	$NiNO_3 \cdot 6H_2O$ /mmol	$Na_2MoO_4 \cdot 2H_2O$ /mmol	H_2O /ml	CH_3CH_2OH /ml
CNF@NiMo-0.5	0.5	0.5	15	15
CNF@NiMo-1	1	1	15	15
CNF@NiMo-2	2	2	15	15
CNF@NiMo-3	3	3	15	15

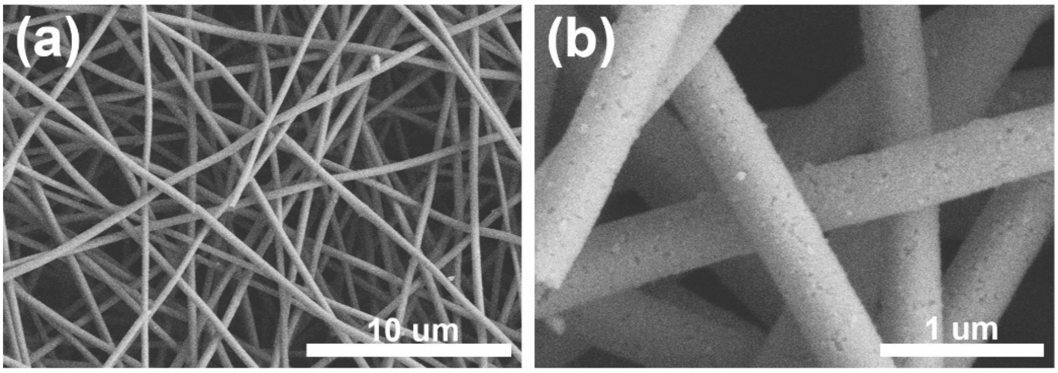

Figure S1. Typical SEM images of carbon nanofibers: (a) low magnification; (b) high magnification

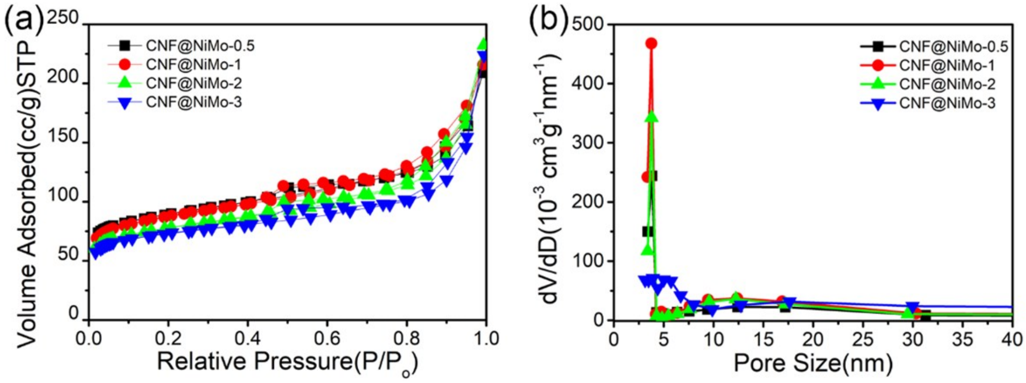

Figure S2. (a) N_2 adsorption-desorption isotherms of CNF@NiMo-0.5, CNF@NiMo-1, CNF@NiMo-2, CNF@NiMo-3 and (b) the corresponding pore size distributions

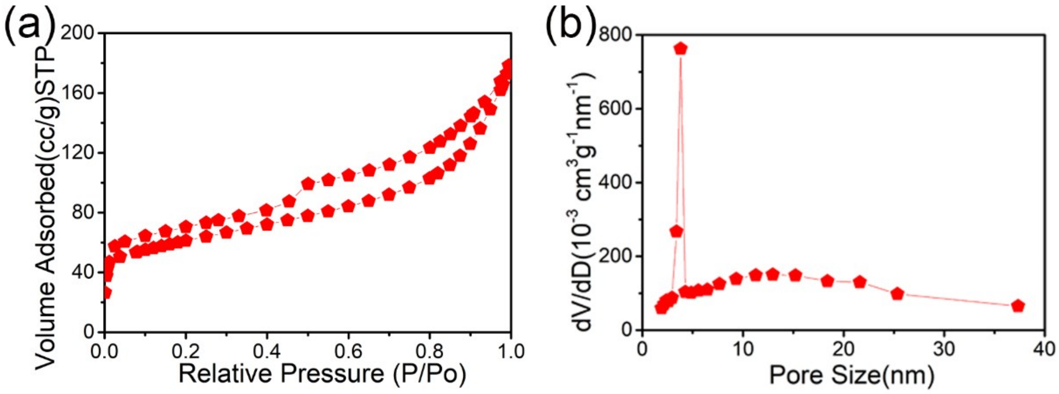

Figure S3. (a) N_2 adsorption-desorption isotherms of pure CNFs before $NiMoO_4$ grown and (b) the corresponding pore size distributions

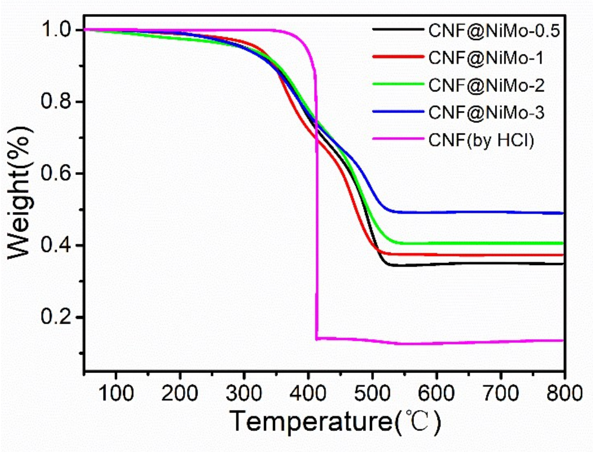

Figure S4. TGA curves of the CNF@NiMo-0.5, CNF@NiMo-1, CNF@NiMo-2, CNF@NiMo-3 and CNFs

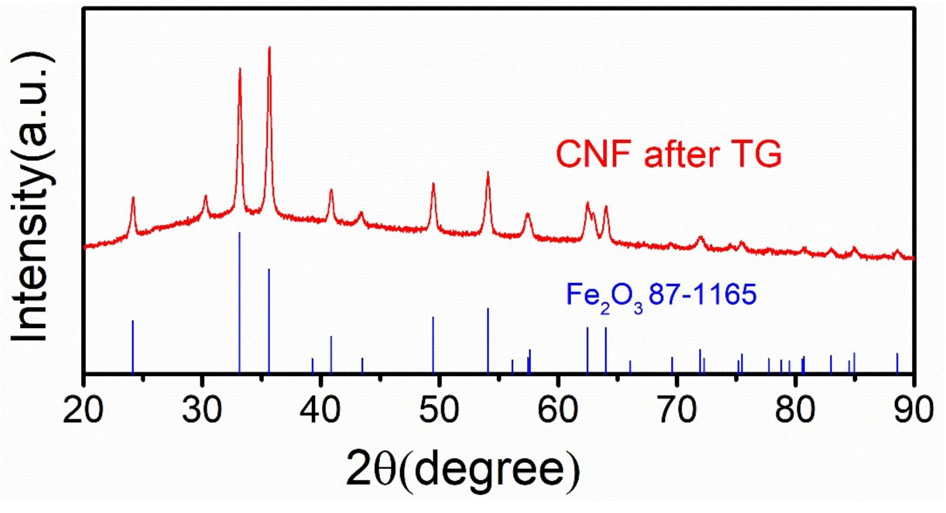

Figure S5. XRD pattern of the TGA residue

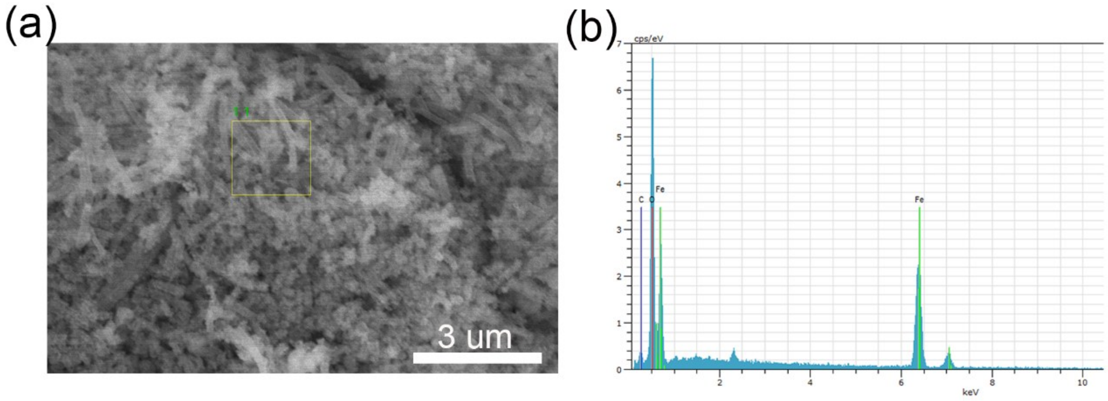

Figure S6. The characterization of TGA residue, (a) SEM image and (b) the corresponding EDS spectroscopy

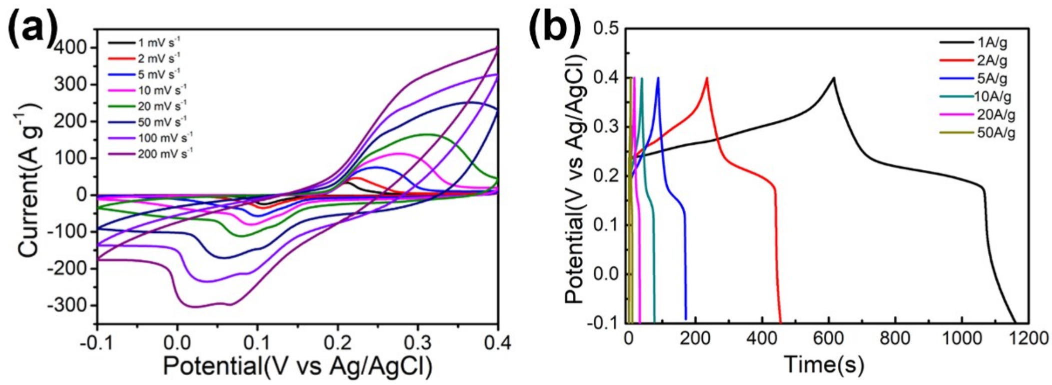

Figure S7. (a) the CV curves and (b) the Galvanostatic charge/discharge voltage profiles of pure $NiMoO_4$ synthesized by the same procedure with CNF@NiMo-2.

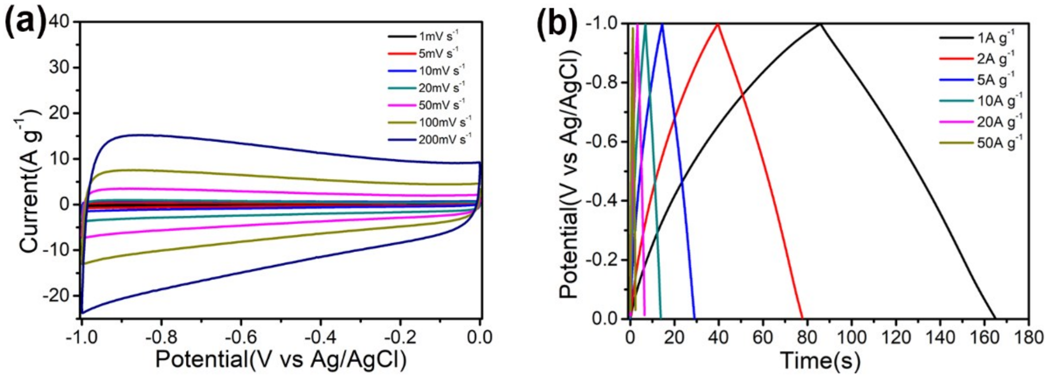

Figure S8. (a) the CV curves and (b) the Galvanostatic charge/discharge voltage profiles of pristine CNFs electrode

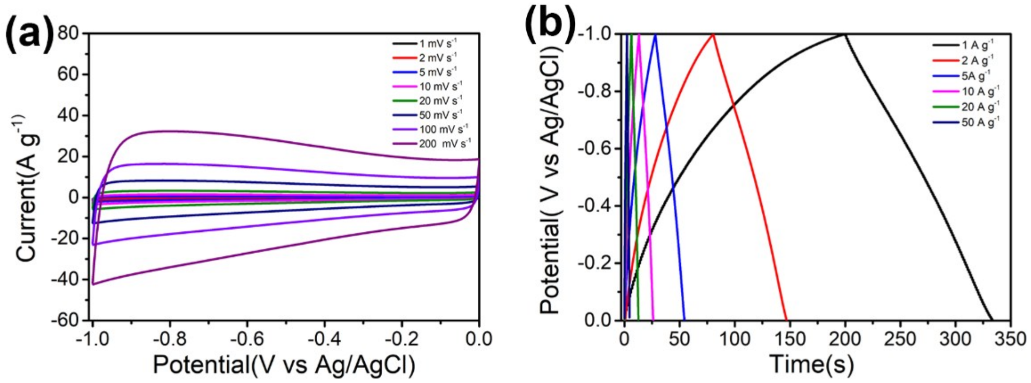

Figure S9. (a) the CV curves and (b) the Galvanostatic charge/discharge voltage profiles of activated carbon.

Table S2 Summary of the reported $NiMoO_4$ based materials (in term of the specific surface area, the specific capacitance and the rate capability)

**Materials**	**Specific Surface Area**	**Specific Capacity (1 A g^{-1})**	**Rate Capability (20 A g^{-1})**	**Reference**
$NiMoO_4$ Nanospheres	58.2 m^2 g^{-1}	974 F g^{-1}	84% 10 A g^{-1}	[1]
$NiMoO_4$ Nanosheet/NF	79 m^2 g^{-1}	1221 F g^{-1}	79% 20 A g^{-1}	[2]
$NiMoO_4 \cdot H_2O$ nanoflake/NF	14.1 m^2 g^{-1}	1300 F g^{-1}	73% 10 A g^{-1}	[3]
$NiMoO_4$ nanosheets	107.4 m^2 g^{-1}	1200 F g^{-1}	<75% 20 A g^{-1}	[4]
$NiMoO_4 \cdot H_2O$ nanotubes	128.5 m^2 g^{-1}	864 F g^{-1}	70% 4 A g^{-1}	[5]
$NiMoO_4$ nanorod/rGO	50.8 m^2 g^{-1}	1274 F g^{-1}	45% 10 A g^{-1}	[6]
**CNF@NiMo-2**	**283 m^2 g^{-1}**	**1840 F g^{-1}**	**81% 10 A g^{-1}**<br>**78% 20 A g^{-1}**	**This Work**

References

[1] D. Cai, D. Wang, B. Liu, Y. Wang, Y. Liu, L. Wang, H. Li, H. Huang, Q. Li, T. Wang, ACS applied materials & interfaces 5 (2013) 12905.

[2] S. Peng, L. Li, H.B. Wu, S. Madhavi, X.W. Lou, Advanced Energy Materials 5 (2015) 1401172.

[3] C. Qing, Y. Liu, X. Sun, X. OuYang, H. Wang, D. Sun, B. Wang, Q. Zhou, L. Xu, Y. Tang, RSC Advances 6 (2016) 67785.

[4] D. Cai, B. Liu, D. Wang, Y. Liu, L. Wang, H. Li, Y. Wang, C. Wang, Q. Li, T. Wang, Electrochimica Acta 115 (2014) 358.

[5] Z. Yin, S. Zhang, Y. Chen, P. Gao, C. Zhu, P. Yang, L. Qi, Journal of Materials Chemistry A 3 (2015) 739.

[6] T. Liu, H. Chai, D. Jia, Y. Su, T. Wang, W. Zhou, Electrochimica Acta 180 (2015) 998.
